# Correction: The recycling endosome protein Rab25 coordinates collective cell movements in the zebrafish surface epithelium

**DOI:** 10.7554/eLife.79841

**Published:** 2022-05-19

**Authors:** Patrick Morley Willoughby, Molly Allen, Jessica Yu, Roman Korytnikov, Tianhui Chen, Yupeng Liu, Isis So, Haoyu Wan, Neil Macpherson, Jennifer A Mitchell, Rodrigo Fernandez-Gonzalez, Ashley EE Bruce

**Keywords:** Zebrafish

Willoughby PM, Allen M, Yu J, Korytnikov R, Chen T, Liu Y, So I, Macpherson N, Mitchell JA, Fernandez-Gonzalez R, Bruce AEE. 2021. The recycling endosome protein Rab25 coordinates collective cell movements in the zebrafish surface epithelium . *eLife* **10**:e66060. doi: 10.7554/eLife.66060.

Published 23 March 2021

After publication we became aware that an earlier version of the Materials and methods section, which was unedited and incomplete, was inadvertently included in the final draft. In addition, a supplemental analysis used three plasmids to disrupt actin specifically in the yolk cell. These plasmids were incorrectly referenced to a previous publication from the lab, but they have not been published. Haoyu Wan generated the plasmids and provided key input on the experimental design for the data presented in Figure 2—figure supplement 2B. We are therefore formally correcting the eLife paper to include Haoyu Wan as a co-author on this paper. Haoyu Wan has reviewed the manuscript and supports the conclusions and agrees to be responsible for all parts of the paper in its published form. These omissions do not impact any of the results reported in the paper.

We have corrected the manuscript as follows:

#1

Original author list:

Willoughby PM, Allen M, Yu J, Korytnikov R, Chen T, Liu Y, So I, Macpherson N, Mitchell JA, Fernandez-Gonzalez R, Bruce AEE.

Corrected author list:

Willoughby PM, Allen M, Yu J, Korytnikov R, Chen T, Liu Y, So I, Wan H, Macpherson N, Mitchell JA, Fernandez-Gonzalez R, Bruce AEE.

Details for omitted author:

Haoyu Wan

Department of Cell and Systems Biology

University of Toronto, Toronto, Canada

Contribution: Resources, methodology, Writing – reviewing and editing

Competing interests: None

#2

Original section of text from the Results:

Rab25a and Rab25b are required for normal epiboly movements

To explore the functions of Rab25a and Rab25b, CRISPR/Cas9 gene editing was used to generate maternal-zygotic (MZ) mutant lines. Guide RNAs were designed to target exon two which encodes the GTPase domain, the functional domain of Rab proteins (Mitra et al., 2017). We characterized two *rab25a* mutant alleles from two founder fish, a 13-base pair (bp) deletion (2.3) and 29 bp insertion (4).

Corrected text:

Rab25a and Rab25b are required for normal epiboly movements

To explore the functions of Rab25a and Rab25b, CRISPR/Cas9 gene editing was used to generate maternal-zygotic (MZ) mutant lines. Guide RNAs were designed to target exon two which encodes the GTPase domain, the functional domain of Rab proteins (Mitra et al., 2017). We characterized two rab25a mutant alleles from two founder fish, a 13-base pair (bp) deletion (2.3) and a 24 bp insertion / 3 bp deletion (4).

#3

Original section of text from Materials and Methods:

CRISPR/Cas9 mutant generation

To determine Cas9 target sites against rab25a and rab25b (ZFIN ID: ZDB-Gene-041212–69; ZDB-Gene-050706–113), the CHOPCHOP tool was used (https://chopchop-cbu-uib-no.myaccess.library.utoronto.ca).

Corrected text:

To determine Cas9 target sites against rab25a and rab25b (ZFIN ID: ZDB-Gene-041212–69; ZDB-Gene-050706–113), the CHOPCHOP tool was used (https://chopchop.cbu.uib.no)

#4

Original section of text from Materials and Methods (CRISPR/Cas9 Mutant Generation):

The Ambion MegaScript T7 kit was used to transcribe sgRNA in vitro. gRNA (50 pg) was coinjected with *cas9* mRNA (300 pg) (pT3TS-nCas9 [Xba1 digest] Addgene plasmid: #46,757 Jao et al., 2013; transcribed using mMESSAGE mMachine T3 kit Life technologies [AM1348]).

Corrected text:

The Ambion MegaScript T7 kit was used to transcribe sgRNA in vitro. gRNA (50 pg) was coinjected with *cas9* mRNA (300 pg)(pT3TS-nCas9 [Xba1 digest] Addgene plasmid: #46,757 Jao et al., 2013; transcribed using mMESSAGE mMachine T3 kit Life technologies [AM1348]). The Zebrafish Genetics and Disease Models Core Facility at the Hospital for Sick Children generated founder Rab25a CRISPR zebrafish using the guide RNA that we designed.

#5

Original section of text from Materials and Methods (CRISPR/Cas9 Mutant Generation):

This led to the identification of two *rab25a* mutations from two different founder fish and one

*rab25b* mutation: rab25a^2.3^–13 BP Deletion 5’-TTGCTTTCAGTGGTTTTAATTGGAGAA———————————
AG-3’ rab25a^4^- 29 BP Insertion
5’TTGCTTTCAGTGGTTTTAATTGGAGAATTGGAAAGTTGGAAAGCGCAACTTTGGG
TTGGGTTGGAAAG-3’ rab25b- 9 BP Deletion 5’-TG———————CCATCACCTCTGCG
TGAGTTTG-3’

Corrected Text:

This led to the identification of two *rab25a* mutations from two different founder fish and one

*rab25b* mutation: rab25a^2.3^–13 bp deletion 5’-TTGCTTTCAGTGGTTTTAATTGGAGAA———————————
AG-3’ rab25a^4^ – 24 bp insertion / 3 bp deletion
5’TTGCTTTCAGTGGTTTTAATTGGAGAATTGGAAAGTTGGAAAGCGCAACTTTGGG
TTGGGTTGGAAAG-3’ rab25b –18 bp deletion 5’-TG———————CCATCACCTCTGCG
TGAGTTTG-3’

#6

Original section of text from Materials and Methods (Cloning): *rab25b* PCR products were gel extracted and recombined with pDONR221 using BP clonase. Clones were validated by sequencing. Fusion proteins and transgenic vectors were generated by gateway recombination using LR Clonase.

Corrected text:

Rab25b fusion proteins

To generate Rab25b fusion proteins, forward and reverse primers containing *attB1* and *attB2* BP Clonase recognition arm sequences were used to PCR amplify *rab25b. attb*-tagged *rab25b* PCR Products were gel extracted and recombined into a pDONR221 entry vector using BP Clonase (Invitrogen 11789013). Clones were identified via kanamycin selection and validated by sequencing.

*attB1rab25b* Forward Primer: 5’GGGGACAAGTTTGTACAAAAAAGCAGGCTTCATGGGGTCTGATGAGGCCTA-3’
*attB2rab25b* Reverse Primer: 5’GGGGACCACTTTGTACAAGAAAGCTGGGTGTCACAAGTTTTTACAGCAGG-3’

pDONR221-*rab25b* vectors were recombined into a pCS2 +SP6 promoter destination vector by Gateway LR Clonase reaction (Invitrogen 11791020). Destination vectors contained a 5’ SP6 promoter sequence, followed by either eGfp or mCherry with Rab25 integrated in the 5’ to 3’ direction downstream, resulting in a N-terminally tagged Rab25b fusion protein. Sequencing was used to confirm integration and reading frame.

#7

**Original section of text from Materials and Methods (Cloning):** Text missing

Corrected Text:

FP2 Constructs

The constructs pFP2-DeAct-SpvB, pFP2-DeAct-GS1 and pFP2-DN-RhoA were generated to block actin polymerization/contraction in the yolk cell. pCMV-DeAct-SpvB (Addgene plasmid 89446) and pCMV-DeAct-GS1 (Addgene plasmid 89445) were gifts from Bradley Zuchero (Harterink et al., 2017) and pFP2 was kindly provided by Arne Lekven (Narayanan and Lekven, 2012). Both DeActs were PCR amplified and cloned into pCS2+. SpvB was amplified using primers containing Cla1/Stu1 restriction sequences for forward and reverse primers, respectively. PCR fragments were digested with either Cla/Stu1, and ligated into pCS2+. GS1 was amplified using primers containing BamHI/StuI restriction sequences for forward and reverse primers, respectively. PCR fragments were digested with either BamHI/StuI, and ligated into pCS2+. SpvB, GS1 and DN-Rho were cloned from pCS2 +into pFP2 using the Gibson assembly method (Gibson, et al. 2009).

Primers used to amplify DeActs were:

SpvB Forward primer:
5’- ACGATCGATGCCACCATGGGAGGTAATTCATCTCG-3’
SpvB Reverse primer:
5’-GACAGGCCTTCATGAGTTGAGTACCCTCA-3’.
GS1 Forward primer:
5’-ACGGGATCCGCCACCATGGTGGTGGAACACCCCGA-3’
GS1 Reverse primer:
5’-CCGAGGCCTTCAGAATCCTGATGCCACAC-3’.

Primers used to clone DeActs and DN-RhoA from pCS2 +into pFP2 were:

Forward primer:
5’-GGTCACTCACGCAACAATACAAGCTACTTGTTCTTTTTG-3’
Reverse primer:
5’-CATGTCTGGATCATCATTACGTAATACGACTCACTATAG-3’

#8

**Original section of text from Materials and Methods:** Text missing

Corrected text:

Transgenic Lines

Rab25b Transgenic rescue line

To generate a transgenic rescue construct for Rab25b, forward and reverse primers containing *attB1* and *attB2* BP Clonase recognition arm sequences were used to PCR amplify *rab25b. attb*-tagged *rab25b* PCR Products were gel extracted and recombined into a pDONR221 entry vector using BP Clonase (Invitrogen 11789013). Clones were identified via kanamycin selection and validated by sequencing.

*attB1rab25b* Forward Primer: 5’GGGGACAAGTTTGTACAAAAAAGCAGGCTTCATGGGGTCTGATGAGGCCTA-3’
*attB2rab25b* Reverse Primer: 5’GGGGACCACTTTGTACAAGAAAGCTGGGTGTCACAAGTTTTTACAGCAGG-3’

pDONR221-*rab25b* vectors were recombined into a Tol2 transgenic destination vector by Gateway LR Clonase reaction (Invitrogen 11791020). Destination transgenic vectors contained two Tol2 recognition sequences that flanked divergent ß-actin and *myl7* promoter sequences. Rab25b was integrated in the 5’ to 3’ orientation downstream of the ß-actin promoter sequence. Sequencing was used to confirm integration and reading frame. The *myl7* promoter sequences contained a downstream RFP expression cassette for screening purposes. To generate Tg(*actb1:rab25b,myl7:RFP*) fish, Tol2 mRNA (25 pg) and the destination vector *Tol2-RFP-myl7*:ß-actin-*rab25b-Tol2* (50 pg) were injected into 1 cell stage embryos. Embryos were screened at 48 hpf to confirm Myl7 heart restricted fluorescence and grown to adulthood.

Rab25a Myosin Transgenic Line

Female Tg(*actb2:myl12.1-eGFP*) fish were crossed to *rab25a*^4^ homozygous males. Heterozygous transgenic embryos were screened for fluorescence at 24hpf and grown to adulthood. Tg:(*actb2:myl12.1-eGFP*, *rab25a^4^* (+/-)) fish were in-crossed to generate Tg:(*actb2:myl12.1-eGFP, rab25a^4^* (-/-)) adult fish, which were screened for fluorescence at 24hpf and genotyped for the *rab25a* mutation by PCR/Hinf1 restriction digest.

#9

Original section of text from Materials and Methods:

Whole-mount immunohistochemistry

Antibody staining was performed as previously described (Lepage, 2014). Dilutions were as follows: rhodamine-phalloidin (1:200), anti-E-cadherin (Abcam, 1:1000), anti-ZO-1 (1:500), anti-phospho-myosin-light chain 2 Ser 19 (cell signaling, 1:100). Embryos were mounted in either 80% glycerol or 0.05% low-melt agarose. Secondary antibodies used were goat-anti-mouse Alexa 488 (Invitrogen, 1:500) and goat anti-rabbit-Cy3 (Jackson immunoresearch,1:500). Sytox green (Invitrogen) was dilution to 0.5 mM in fixative.

Corrected Text:

Whole-mount immunohistochemistry

Antibody staining was performed as previously described (Lepage, 2014). Dilutions were as follows: rhodamine-phalloidin (1:200), anti-E-cadherin (Abcam, 1:1000), anti-RAB11B (Abcam 1:200), anti-ZO-1 (ThermoFisher Scientific, 1:500), anti-phospho-myosin-light chain 2 Ser 19 (Cell Signaling, 1:100). Embryos were mounted in either 80% glycerol or 0.05% low-melt agarose. Secondary antibodies used were goat-anti-mouse Alexa 488 (Invitrogen A11001, 1:500), goat anti-rabbit Alexa 488 (Invitrogen A11008, 1:500), goat anti-rabbit-Cy3 (Jackson ImmunoResearch AB-2338006,1:500). Sytox green (Invitrogen) was diluted to 0.5 mM in fixative. For embryos co-labelled with phalloidin and antibody, phalloidin was incubated at 1:200 dilution with primary antibody overnight at 4 °C in block solution.

#10

As a result of omissions in the Material and methods section, some key resources were omitted from the table and some information was incomplete.

Original Key Resources Table:

**Table:** 

Reagent type (species) or resource	Designation	Source or reference	Identifiers	Additional information
chemical compound	rhodamine-phalloidin	Invitrogen	Cat #R415	(1:200)
chemical compound	sytox green	Invitrogen	Cat #S7020	(1:1000)
chemical compound	pHrodo Red Dextran	Invitrogen	Cat# P10361	(1 mg/ml)
antibody	anti-phospho-myosin-light chain 2 Ser 19 (Rabbit polyclonal)	Cell Signalling	Cat #3,671	IF(1:100)
antibody	anti-Cdh1 (Rabbit polyclonal)	AnaSpec	Cat# 55,527 s	IF (1:1000)
commercial assay or kit	MEGAscript T7-Transcription Kit	Ambion	Cat#AMB1334	
commercial assay or kit	MegaClear Clean Up Kit	Ambion	Cat#AM1908	
commercial assay or kit	mMessage mMachine T3 Transcription Kit	ThermoFisher Scientific	Cat#AM1348	
commercial assay or kit	NucAway Spin Column	Ambion	Cat#AM10070	
strain (*D. rerio*)	AB wildtype	Zebrafish International Resource Centre	RRID:BDSC_5138	
strain (*D. rerio*)	Tg:(XIEef1a1:dclk2DeltaK-GFP)	Sepich et al., 2011		
strain (*D. rerio*)	Tg:(XIEef1a:eGFP-tubα8I)	Fei, et al, 2019		
strain (*D. rerio*)	Tg:(actb2:myl12.1-eGFP)	Maitre et al., 2012		
strain (*D. rerio*)	MZrab25a^4^	This study	https://zfin.org/ZDB-ALT-201221-11	
strain (*D. rerio*)	MZrab25a^2.3^	This study	https://zfin.org/ZDB-ALT-201221-10	
strain (*D. rerio*)	MZrab25b	This study	https://zfin.org/ZDB-ALT-201221-12	
strain (*D. rerio*)	Tg(*actb1:rab25b*)	This study		
strain (*D. rerio*)	MZrab25a^4^ Tg:(actb1:myl12.1-eGFP)	This study		
strain (*D. rerio*)	MZrab25b Tg:(actb1:myl12.1-eGFP)	This study		
sequence-based reagent	rab25a_F	This study	PCR Primers	Forward: TATTTATTCACCAAGCGGTTG (for genotyping)
sequence-based reagent	rab25a_R	This study	PCR Primers	Reverse: GAGTGGTTCTGGGTGTGAGTC (for genotyping)
sequence-based reagent	rab25b_F	This study	PCR Primers	Forward: TGTTTGCAGTGGTTCTTATTGGAG (for genotyping)
sequence-based reagent	rab25b_R	This study	PCR Primers	Reverse: ATTACGTTCGCTTGCAGAATTT (for genotyping)
sequence-based reagent	rab25a_F	This study	PCR Primers	Forward: ATGGGGACAGATTTAGCCTACAAC (for cDNA)
sequence-based reagent	rab25a_R	This study	PCR Primers	Reverse: CGAAGCTGCTGCAAAAACTCCTGA (for cDNA)
sequence-based reagent	rab25a_F	This study	PCR primers	Forward: GGATCCATGGGGACAGATTTAGCCTACAAC (ligation into pCS2 +via restriction digest via BamH1, Xho1)
sequence-based reagent	rab25a_R	This study	PCR primers	Reverse: CTGGAGCGAAGCTGCTGCAAAAACTCCTGA (ligation into pCS2 +via restriction digest via BamH1, Xho1)
sequence-based reagent	rab25a_F	This study	PCR Primers	Forward: CTCGAGGGCGCCACCATGGGGACAGATTTAGCCTACAAC (ligation with into pCS2 +*venus* via Xho1 restriction digest)
sequence-based reagent	rab25a_R	This study	PCR Primers	Reverse: CTCGAGCGAAGCTGCTGCAAAAACTCCTGA (ligation with into pCS2 +*venus* via Xho1 restriction digest)
sequence-based reagent	rab25b_F	This study	PCR Primers	Forward: GGGGACAAGTTTGTACAAAAAAGCAGGCT TCATGGGGTCTGATGAGGCCTA (*rab25b* with *attb1* for recombination into pDONR221)
sequence-based reagent	rab25b_R	This study	PCR Primers	Reverse: GGGGACCACTTTGTACAAGAAAGCTGGGT GTCACAAGTTTTTACAGCAGG (*rab25b* with *attb1* for recombination into pDONR221)
sequence-based reagent	rab11a_F	This study	PCR Primers	Forward: AGAAAAACGGTCTGTCCTTC (qPCR)
sequence-based reagent	rab11a_R	This study	PCR Primers	Reverse: TCAGGATGGTCTGAAAAGCA (qPCR)
sequence-based reagent	rab25a_F	This study	PCR Primers	Forward: GAAGTGACCAGAGGCTCGAT (qPCR)
sequence-based reagent	rab25a_R	This study	PCR Primers	Reverse: GGAGTTTTTGCAGCAGCTT (qPCR)
sequence-based reagent	rab25b_F	This study	PCR Primers	Forward: TCGGAGCTCTGCTGGTTTAT (qPCR)
sequence-based reagent	rab25b_R	This study	PCR Primers	Reverse: GCGTGATCGTAGAGCTCCTT (qPCR)
sequence-based reagent	Lsm12_F	This study	PCR Primers	Forward: AGTTGTCCCAAGCCTATGCAATCAG (qPCR)
sequence-based reagent	Lsm12_R	This study	PCR Primers	Reverse: CCACTCAGGAGGATAAAGACGAGTC (qPCR)
recombinant DNA reagent	pCS2+ (plasmid)	Rupp et al., 1994		SP6/T7 based backbone
recombinant DNA reagent	pCS2 +egfp-rab25b (plasmid)	This study		egfp-Rab25b version of pCS2+
recombinant DNA reagent	pCS2 +mcherry-rab25b (plasmid)	This study		mcherry-rab25b version of pCS2+
recombinant DNA reagent	pCS2 +venus-rab25a (plasmid)	This study		venus-rab25a version of pCS2+
recombinant DNA reagent	pCS2+-mcherry-Rab11a (plasmid)	Rathbun et al. ,2020		mcherry-Rab11a version of pCS2+
recombinant DNA reagent	pCS2+-mcherry-Mklp1 (plasmid)	Rathbun et al., 2020		mCherry-Mklp1 version of pCS2+
recombinant DNA reagent	pCS2 +lyn-eGfp	A gift from Brian Ciruna		lyn-eGfp version of pCS2+
recombinant DNA reagent	pTol2-actb1	A gift from Brian Ciruna		Tol2 transgenics
recombinant DNA reagent	pTol2-actb1:rab25b	This study		rab25b version of pTol2-actb1
recombinant DNA reagent	pCS2+- Gfp-Utrophin	Addgene	RRID: Addgene_26737	Gfp-Utrophin version of pCS2+
recombinant DNA reagent	pFP2	Narayanan et al., 2012		*Wnt8a* enhancer based backbone
recombinant DNA reagent	pFP2-GS1	Fei et al, 2019		GS1 version of FP2
recombinant DNA reagent	pFP2-SPVB	Fei et al, 2019		SPVB version of FP2
recombinant DNA reagent	pFP2-DN-RhoA	This study		DN-RhoA version of FP2
recombinant DNA reagent	pT3TS-nCas9	Jao et al., 2013	RRID: Addgene_46757	T3 based backbone

Corrected Key Resources Table:

**Table:** 

Reagent type (species) or resource	Designation	Source or reference	Identifiers	Additional information
chemical compound	rhodamine-phalloidin	Invitrogen	Cat #R415	(1:200)
chemical compound	sytox green	Invitrogen	Cat #S7020	(1:1000)
chemical compound	pHrodo Red Dextran	Invitrogen	Cat# P10361	(1 mg/ml)
antibody	anti-phospho-myosin-light chain 2 Ser 19 (Rabbit polyclonal)	Cell Signalling	Cat #3,671	IF(1:100)
antibody	anti-Cdh1 (Rabbit polyclonal)	AnaSpec	Cat# 55,527 s	IF (1:1000)
antibody	anti-RAB11B (Rabbit polyclonal)	Abcam	Cat#3,612	IF (1:200)
antibody	anti-ZO-1	ThermoFisher Scientific	Cat#33–9100 ZO1-1A12	IF (1:500)
commercial assay or kit	MEGAscript T7-Transcription Kit	Ambion	Cat#AMB1334	
commercial assay or kit	MegaClear Clean Up Kit	Ambion	Cat#AM1908	
commercial assay or kit	mMessage mMachine T3 Transcription Kit	ThermoFisher Scientific	Cat#AM1348	
commercial assay or kit	NucAway Spin Column	Ambion	Cat#AM10070	
strain (*D. rerio*)	AB wildtype	Zebrafish International Resource Centre	RRID:BDSC_5138	
strain (*D. rerio*)	Tg:(XIEef1a1:dclk2DeltaK-GFP)	Sepich et al., 2011	https://zfin.org/ZDB-TGCONSTRCT-090702-3	
strain (*D. rerio*)	Tg:(XIEef1a:eGFP-tubα8I)	Fei, et al, 2019	https://zfin.org/ZDB-ALT-170215-4	Line: uot3Tg
strain (*D. rerio*)	Tg:(actb2:myl12.1-eGFP)	Maitre et al., 2012	https://zfin.org/ZDB-ALT-130108-2	Line: e2212Tg
strain (*D. rerio*)	MZrab25a^4^	This study	https://zfin.org/ZDB-ALT-201221-11	Line: uot10
strain (*D. rerio*)	MZrab25a^2.3^	This study	https://zfin.org/ZDB-ALT-201221-10	Line: uot9
strain (*D. rerio*)	MZrab25b	This study	https://zfin.org/ZDB-ALT-201221-12	Line: uot11
strain (*D. rerio*)	Tg(*actb1:rab25b,myl7:RFP*)	This study	https://zfin.org/ZDB-ALT-220311-4	Line: uot16Tg
strain (*D. rerio*)	MZrab25a^4^ Tg:(actb1:myl12.1-eGFP)	This study		
strain (*D. rerio*)	MZrab25b Tg:(actb1:myl12.1-eGFP)	This study		
sequence-based reagent	rab25a_F	This study	PCR Primers	Forward: TATTTATTCACCAAGCGGTTG (for genotyping)
sequence-based reagent	rab25a_R	This study	PCR Primers	Reverse: GAGTGGTTCTGGGTGTGAGTC (for genotyping)
sequence-based reagent	rab25b_F	This study	PCR Primers	Forward: TGTTTGCAGTGGTTCTTATTGGAG (for genotyping)
sequence-based reagent	rab25b_R	This study	PCR Primers	Reverse: ATTACGTTCGCTTGCAGAATTT (for genotyping)
sequence-based reagent	rab25a_F	This study	PCR Primers	Forward: ATGGGGACAGATTTAGCCTACAAC (for cDNA)
sequence-based reagent	rab25a_R	This study	PCR Primers	Reverse: CGAAGCTGCTGCAAAAACTCCTGA (for cDNA)
sequence-based reagent	rab25a_F	This study	PCR primers	Forward: GGATCCATGGGGACAGATTTAGCCTACAAC (ligation into pCS2 +via restriction digest via BamH1, Xho1)
sequence-based reagent	rab25a_R	This study	PCR primers	Reverse: CTGGAGCGAAGCTGCTGCAAAAACTCCTGA (ligation into pCS2 +via restriction digest via BamH1, Xho1)
sequence-based reagent	rab25a_F	This study	PCR Primers	Forward: CTCGAGGGCGCCACCATGGGGACAGATTTAGCCTACAAC (ligation with into pCS2 +*venus* via Xho1 restriction digest)
sequence-based reagent	rab25a_R	This study	PCR Primers	Reverse: CTCGAGCGAAGCTGCTGCAAAAACTCCTGA (ligation with into pCS2 +*venus* via Xho1 restriction digest)
sequence-based reagent	rab25b_F	This study	PCR Primers	Forward: GGGGACAAGTTTGTACAAAAAAGCAGGCT TCATGGGGTCTGATGAGGCCTA (*rab25b* with *attb1* for recombination into pDONR221)
sequence-based reagent	rab25b_R	This study	PCR Primers	Reverse: GGGGACCACTTTGTACAAGAAAGCTGGGT GTCACAAGTTTTTACAGCAGG (*rab25b* with *attb1* for recombination into pDONR221)
sequence-based reagent	rab11a_F	This study	PCR Primers	Forward: AGAAAAACGGTCTGTCCTTC (qPCR)
sequence-based reagent	rab11a_R	This study	PCR Primers	Reverse: TCAGGATGGTCTGAAAAGCA (qPCR)
sequence-based reagent	rab25a_F	This study	PCR Primers	Forward: GAAGTGACCAGAGGCTCGAT (qPCR)
sequence-based reagent	rab25a_R	This study	PCR Primers	Reverse: GGAGTTTTTGCAGCAGCTT (qPCR)
sequence-based reagent	rab25b_F	This study	PCR Primers	Forward: TCGGAGCTCTGCTGGTTTAT (qPCR)
sequence-based reagent	rab25b_R	This study	PCR Primers	Reverse: GCGTGATCGTAGAGCTCCTT (qPCR)
sequence-based reagent	SpvB-F	This study	PCR primers	Forward: ACGATCGATGCCACCATGGGAGGTAATTCATCTCG
sequence-based reagent	SpvB-R	This study	PCR primers	Reverse: GACAGGCCTTCATGAGTTGAGTACCCTCA
sequence-based reagent	GS1-F	This study	PCR primers	ACGGGATCCGCCACCATGGTGGTGGAACACCCCGA
sequence-based reagent	GS1-R	This study	PCR primers	CCGAGGCCTTCAGAATCCTGATGCCACAC
sequence-based reagent	Gibson-F	This study	PCR primers	GGTCACTCACGCAACAATACAAGCTACTTGTTCTTTTTG (for cloning from pCS2 +into pFP2)
sequence-based reagent	Gibson-R	This study	PCR primers	CATGTCTGGATCATCATTACGTAATACGACTCACTATAG (for cloning from pCS2 +into pFP2)
sequence-based reagent	Lsm12_F	This study	PCR Primers	Forward: AGTTGTCCCAAGCCTATGCAATCAG (qPCR)
sequence-based reagent	Lsm12_R	This study	PCR Primers	Reverse: CCACTCAGGAGGATAAAGACGAGTC (qPCR)
recombinant DNA reagent	pCS2+ (plasmid)	Rupp et al., 1994		SP6/T7 based backbone
recombinant DNA reagent	pCS2 +egfp-rab25b (plasmid)	This study		egfp-Rab25b version of pCS2+
recombinant DNA reagent	pCS2 +mcherry-rab25b (plasmid)	This study		mcherry-rab25b version of pCS2+
recombinant DNA reagent	pCS2 +venus-rab25a (plasmid)	This study		venus-rab25a version of pCS2+
recombinant DNA reagent	pCS2+-mcherry-Rab11a (plasmid)	Rathbun et al. ,2020		mcherry-Rab11a version of pCS2+
recombinant DNA reagent	pCS2+-mcherry-Mklp1 (plasmid)	Rathbun et al., 2020		mCherry-Mklp1 version of pCS2+
recombinant DNA reagent	pCS2 +lyn-eGfp	A gift from Brian Ciruna		lyn-eGfp version of pCS2+
recombinant DNA reagent	pTol2-actb1	A gift from Brian Ciruna		Tol2 transgenics
recombinant DNA reagent	pTol2-actb1:rab25b	This study		rab25b version of pTol2-actb1
recombinant DNA reagent	pCS2+- Gfp-Utrophin	Addgene	RRID: Addgene_26737	Gfp-Utrophin version of pCS2+
recombinant DNA reagent	pFP2	Narayanan et al., 2012		*Wnt8a* enhancer based backbone
recombinant DNA reagent	pFP2-GS1	This study		GS1 version of FP2
recombinant DNA reagent	pFP2-SPVB	This study		SPVB version of FP2
recombinant DNA reagent	pFP2-DN-RhoA	This study		DN-RhoA version of FP2
recombinant DNA reagent	pT3TS-nCas9	Jao et al., 2013	RRID: Addgene_46757	T3 based backbone
recombinant DNA reagent	pSK-H2B-Rfp:5xUAS:Gfp-Dcx	Distel et al., 2010		

#11

We have identified a typo in Figure 2—figure supplement 1F. The label *sox19* should be *sox17*.

Original Figure 2 - figure supplement 1F:

**Figure 1:**
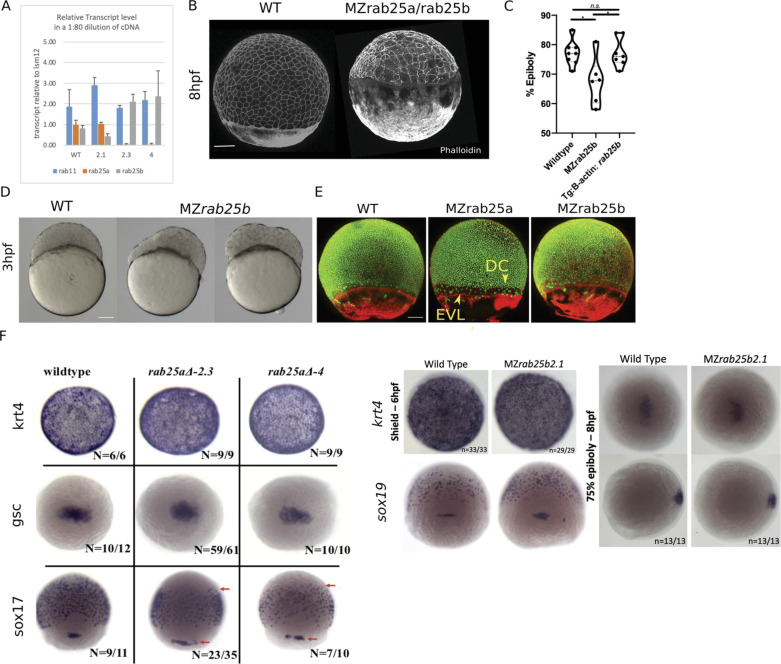


Corrected Figure 2 - figure supplement 1F:

**Figure 2:**
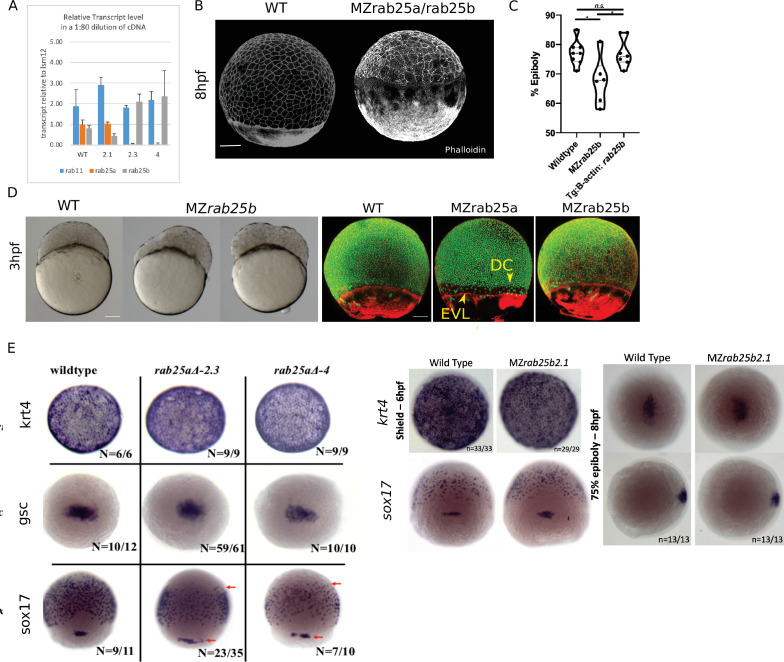


Typo in one label (sox19 instead of sox17)

#12

Additional Citations to be added to the reference section:

Distel M, Hocking JC, Volkmann K, Köster RW. 2010. The centrosome neither persistently leads migration nor determines the site of axonogenesis in migrating neurons in vivo. *Journal of Cell Biology* 19:875–90. doi: 10.1083/jcb.201004154. PMID:21059852.

Gibson DG, Young L, Chuang RY, Venter JC, Hutchison CA 3rd, Smith HO. 2009. Enzymatic assembly of DNA molecules up to several hundred kilobases. *Nature Methods* 6:343–5. doi: 10.1038/nmeth.1318. PMID:19363495.

Harterink M, da Silva ME, Will L, Turan J, Ibrahim A, Lang AE, van Battum EY, Pasterkamp RJ, Kapitein LC, Kudryashov D, Barres BA, Hoogenraad CC, Zuchero JB. 2017. DeActs: genetically encoded tools for perturbing the actin cytoskeleton in single cells. *Nature Methods* 14:479–482. doi: 10.1038/nmeth.4257 PMID:28394337.

The article has been corrected accordingly.

